# Cerebral Amyloid Angiopathy-Related Inflammation (CAA-rI): Three Heterogeneous Case Reports and a Focused Literature Review

**DOI:** 10.3390/brainsci13050747

**Published:** 2023-04-29

**Authors:** Ivo Bozovic, Marta Jeremic, Aleksandra Pavlovic, Carna Jovanovic, Nikola Kresojevic, Nikola Vojvodic, Dejana Jovanovic, Dragoslav Sokic, Milija Mijajlovic

**Affiliations:** 1 Neurology Clinic, University Clinical Center of Serbia, University of Belgrade, 11000 Belgrade, Serbia; ivo.bozovic20@gmail.com (I.B.); marta.jeremic@gmail.com (M.J.); carnajov@gmail.com (C.J.); nikola_kresojevic@yahoo.com (N.K.); nikovojvodic@gmail.com (N.V.); dejana.r.jovanovic@gmail.com (D.J.); dsokic@gmail.com (D.S.); 2 Faculty for Special Education and Rehabilitation, University of Belgrade, 11000 Belgrade, Serbia; aleksandra3003@yahoo.com; 3 Faculty of Medicine, University of Belgrade, 11000 Belgrade, Serbia

**Keywords:** cerebral amyloid angiopathy-related inflammation, cerebral amyloid angiopathy, heterogeneous clinico-radiological presentations, review, patients, diagnostics, treatment

## Abstract

Cerebral amyloid angiopathy-related inflammation (CAA-rI) is a largely reversible, subacute encephalopathy, which is considered as a rare variant of cerebral amyloid angiopathy (CAA). Although the diagnosis of this inflammatory vasculopathy is generally clinico-pathologic, a probable or possible diagnosis can often be established based on current clinico-radiological diagnostic criteria. This is important since CAA-rI is considered as a treatable disorder, which most commonly occurs in the elderly population. Behavioral changes and cognitive deterioration are highlighted as the most common clinical signs of CAA-rI, followed by a heterogeneous spectrum of typical and atypical clinical presentations. However, despite the well-established clinical and radiological features incorporated in the current diagnostic criteria for this CAA variant, this rare disorder is still insufficiently recognized and treated. Here, we have shown three patients diagnosed with probable CAA-rI, with significant heterogeneity in the clinical and neuroradiological presentations, followed by different disease courses and outcomes after the introduction of immunosuppressive treatment. Moreover, we have also summarized up-to-date literature data about this rare, yet underdiagnosed, immune-mediated vasculopathy.

## 1. Introduction

Cerebral amyloid angiopathy-related inflammation (CAA-rI) is a largely reversible, subacute encephalopathy, which is considered as a rare and aggressive variant of cerebral amyloid angiopathy (CAA) [1,2,3,4,5]. The prevalence of CAA increases with age and fits in a range between 30 and 40 cases per 100,000 persons, with most of the sporadic CAA cases diagnosed typically between the 7th and 8th decade of life [1,2,4]. Recent data suggest that the inflammatory variant of CAA commonly occurs earlier (before and in the 7th decade) [1,2,5], but its rare appearance and poor recognition keep the true epidemiologic characteristics of this “young” disease entity still undetermined. CAA-rI is one of the two main pathological entities of inflammatory cerebral amyloid angiopathy (ICAA), an inflammatory response to the amyloid β deposits in small and medium brain vessels [1,3,5,6]. While CAA-rI is considered as a non-destructive perivascular inflammation, the other entity, amyloid β-related angiitis (ABRA), is presented with transmural or intramural inflammation [3,5,6]. Even though the diagnosis of this inflammatory vasculopathy is generally clinico-pathologic, a probable or possible diagnosis can often be established based on current clinico-radiological diagnostic criteria [6,7,8]. Unlike in CAA, the clinical part of the CAA-rI “mosaic” is predominantly characterized by acute or subacute and progressive neuropsychological impairment with or without behavioral changes, but the presence of a severe headache, seizures, and focal neurological deficit is indicative of CAA-rI [1,4,6,8]. Nevertheless, this inflammatory CAA variant is often characterized with the presence of atypical clinical features, which might lead to misdiagnosing and delayed treatment introduction [5,6]. On the other hand, the most suggestive magnetic resonance (MR) imaging findings in CAA-rI are predominantly asymmetric white matter hyperintensities seen on T2 or fluid-attenuated inversion recovery sequences, lobar cerebral microbleeds or cortical superficial siderosis on susceptibility-weighted imaging, and/or signs of lobar intracerebral hemorrhage [1,5,7,9,10]. As previously underlined, an even more significant feature of this infrequent subtype of CAA is a largely satisfying and mostly rapid response to early administered immunosuppressive therapy. However, current treatment recommendations for CAA-rI are mostly based on a case-by-case basis, and small cohorts reports and different approaches are still being evaluated [3]. Moreover, an early treatment response evaluation is mandatory since it can be unsuccessful in more the one-third of the treated patients [3]. However, despite the up-to-date established diagnostic criteria for CAA-rI, this disorder is still insufficiently recognized and treated [1,5]. Therefore, an early and more precise clinico-radiological characterization and recognition of CAA-rI is crucial in order to establish early treatment and improve the prognosis of the disease.

Thus, we have presented three cases of probable CAA-rI with heterogeneous clinical and neuroradiological features, which further underlined the significance of early treatment introduction. Our report was additionally supported with a focused overview of related literature data.

## 2. Case Reports

### 2.1. First Case Description

**Figure 1:**
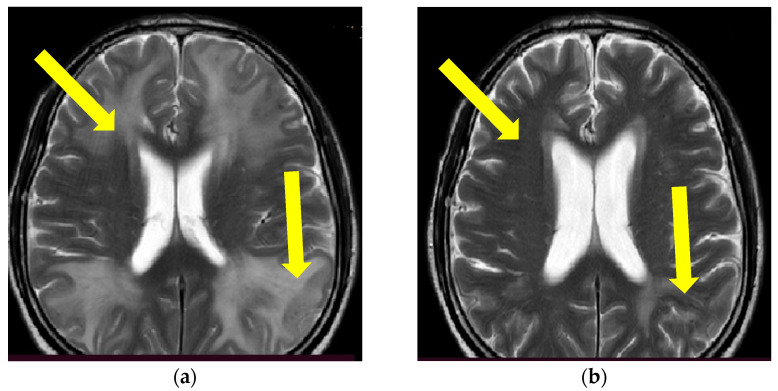
Initial brain MR T2 sequence at admission and before therapy initiation (**a**) and one month after therapy initiation (**b**). Yellow arrows are showing confluent and extensive bilateral periventricular hyperintensities in (**a**) and reduction of hyperintensities of the same locations on (**b**). (Patient No. 1).

**Figure 2:**
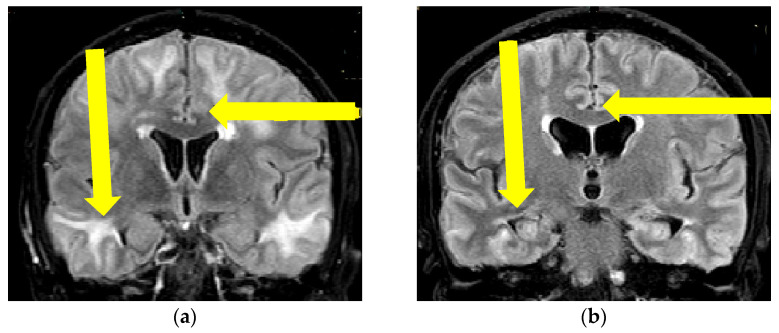
Initial brain MR T2 FLAIR sequence (**a**), and control brain MR examination after immunosuppressive therapy initiation (**b**). Yellow arrows showing the locations of hyperintensities and clear regression of lesions (Patient No. 1).

A 55-year-old man, with a history of essential arterial hypertension, presented with subacute onset of speech disturbance and a discrete right-sided spastic hemiparesis. The initial MR finding was interpreted with the presence of diffuse vasogenic cerebral edema and cerebral microbleeds represented by multiple focal zones of decreased SI on T2* imaging (Figure 1 and Figure 2). He was treated with antihypertensive and anti-edematous therapy, which led to slight clinical improvement. For the initial treatment of the noted hypertensive crisis (TA 190/100 mmHg), an intravenous (IV) bolus of 12.5 mg urapidil was administered followed by sequential reduction of continuous IV doses of urapidil. Oral antihypertensive therapy was also administered, including enalapril (20 mg) and amlodipine (10 mg) until the mean blood pressure of 145/80 mmHg was continuously reached. In addition, 125 mL of 20% IV mannitol four times a day was administered as anti-edematous therapy for five consecutive days. One month later, the patient deteriorated clinically and presented with impaired state of consciousness (level of somnolence, The Glasgow Coma Scale (GCS) 12), spatiotemporal disorientation, mild constant, nonplusing headache, mild right-sided spastic hemiparesis, and self-sufficient but ataxic gait, all of which had developed within 24 h. Except elevated blood pressure (180/120 mmHg), the patient’s other physical examination findings were normal. Standard urine and serum (complete blood count and biochemical) laboratory analyses did not show any significant deviations in the findings. The results of complete immuno-serologic and systemic connective tissue disorder testing were normal (ANA, ANCA, ENA Screen, ANA HEp2, CIC, C3, C4 and VDRL tests).

During the screening for infectious diseases, a chronic hepatitis B viral (HBV) infection was confirmed (verified with positive hepatitis B serologic serum markers and positive real time-polymerase chain reaction (PCR) HBV DNA findings, while all other tests for infectious diseases were negative (HIV ag/at, HBs ag, total HCV antibodies, HSV 1 and 2, CMV, VZV, EBV, and JCV). The screening for autoimmune and paraneoplastic disorders also showed normal results (antibodies against surface antigens of neuronal cells and antineuronal antigens were not detected). Furthermore, chest Rontgen radiation (X-ray) and abdominal and trans-thoracal cardiac ultrasound examinations also showed no findings. The second MR examination showed confluent asymmetric white matter hyperintensities (WMH), predominantly in the bilateral frontal, temporal, and parietooccipital regions, cerebral microbleeds, as well as multiple supratentorial chronic microvascular ischemic lesions and periventricular leukoencephalopathy. A lumbar puncture was performed, and elevated cerebrospinal fluid (CSF) proteins (1.25 g/L) were noted, whilst glycose levels and cell count were normal. Isoelectric focusing of the CSF and serum showed the presence of parallel oligoclonal bands (OCB) in both the CSF and serum. The CSF pathohistological analysis was normal, while the analysis of beta and tau proteins in the CSF did not reveal any findings. All microbiologic and immunological CSF investigations were normal. Genetic analysis of the *Apolipoprotein E* (*ApoE*) and the *NOTCH3* gene were negative in this patient.

Since the patient fulfilled all the criteria for the diagnosis of probable CAA-ri (age > 40 years, decreased consciousness, with the presence of behavioral changes and focal neurologic deficit, MR signs of asymmetric WMH lesions and cerebral microbleeds, with the absence of other possible causes), the patient was initially treated with antihypertensive and anti-edematous therapy, together with IV dexamethasone (8 mg/daily) for 10 days, followed by pulse methylprednisolone therapy (1000 mg/day) for 5 days, after which oral prednisone (1 mg/kg) was slowly tapered down. The patient’s neurological status improved and he showed very discrete hemiparesis and normal gait, and the patient was alert and oriented. A neurological examination was almost without significant impairment and sequelae. A control CSF examination was performed, where normal levels of protein and glycose were detected. A normal cell count was present, and no OCB were registered. Control brain MR showed edema reduction with less extended WMH lesions (Figure 1 and Figure 2). The post treatment neuropsychological examination was without significant impairment.

### 2.2. Second Case Description

**Figure 3:**
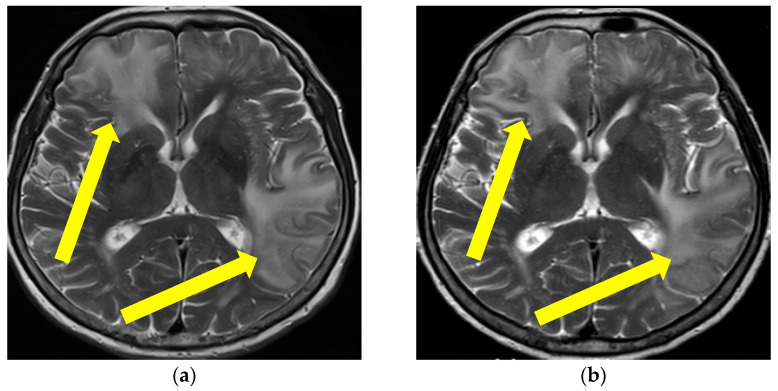
Initial brain MR axial T2W sequence (**a**) and control MR examination three months after therapy initiation (**b**): (**a**) showing bilateral confluent hyperintense lesions, parieto-occipitally and temporally located in the cortex, subcortically and periventricularly, (**b**) shows confluent lesions subcortically, in the left temporal lobe in progression in comparison with previous images one month after therapy initiation. Yellow arrows showing confluent hyperintensities on the right parietally and on the left temporally (Patient No. 2).

**Figure 4:**
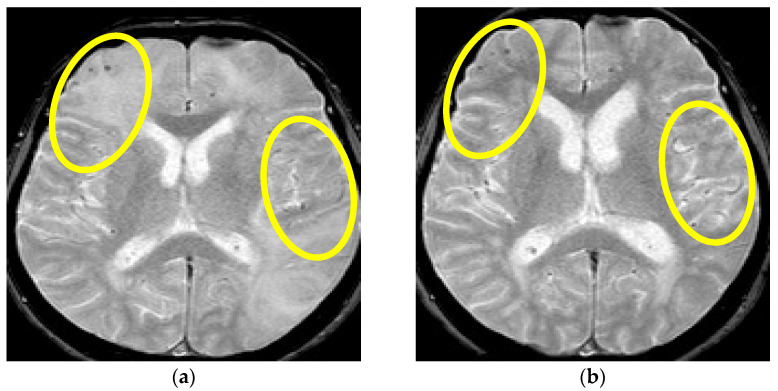
Initial MR axial T2*W sequence (**a**) and control MRI one month after therapy initiation (**b**): (**a**) showing bilateral multiple punctiform hypointensities supratentorially, representing deposits of hemosiderin on the first MR examination, (**b**) showing the persistence of bilateral multiple punctiform hypointensities supratentorially. Yellow circles are showing the bilateral location of the multifocal hypointensities (Patient No. 2).

A 66-year-old female patient, with a history of mild essential arterial hypertension, presented with an episode of transitory aphasia, followed by a severe intensity headache and sudden onset of memory loss. Upon admission, her neurological examination revealed the presence of temporal disorientation with positive frontal release signs, and mild right-sided spastic hemiparesis. Standardized urine and serum (complete blood count, biochemical, hormonal, vitamin, and tumor status, which included also the following complete coagulations status with aPTT, APCR Ratio, LA1, antithrombin, plasminogen, factor XIII, factor X, vWF, factor VII, factor XII, factor XI, factor IX, factor VIII, factor V, factor II, ferritin, folic acid, lactate, ACE, thyroid status with TSH, fT3, fT4 and the following tumor makers CYFRA-21-1, CA 72-4, CEA, CA 19-9, CA 15-3, CA 125) laboratory results were indicative of signs of mild microcytic hypochromic anemia and elevated levels of D-dimer (7.7), while all the other parameters were within the reference range. Immuno-serologic examination showed AMA M2 levels > 1:640 and ANA-HEp2 level of 1:80, while systemic connective tissue disorder testing showed normal findings. Chest X-ray and abdominal ultrasound examination were also normal. Her brain MR examination showed confluent, asymmetrical white matter lesions in both the frontal and temporal lobes, with supratentorial lacunar infarctions, periventricular leukoencephalopathy and cortical microbleeds (Figure 3 and Figure 4), while her MR angiography (MRA) examinations revealed unremarkable findings. The brain positron emission tomography (PET) scan revealed an asymmetric glucose hypometabolism of the bilateral frontal, parietal (left > right), and temporal (left > right) lobes. The cytological and biochemical CSF analysis showed normal cell counts and glucose, but the CSF protein level was elevated (0.83 g/L) while no OCB were present. All microbiologic, immunologic, and sediment CSF investigations were normal. Neuropsychological assessment showed moderate dysexecutive syndrome and episodic memory loss. Finally, the conducted genetic analysis revealed that the patient was a homozygote carrier of *ApoE Epsilon 4* mutation.

Based on these findings a diagnosis of probable CAA-rI was established. Initially, pulse methylprednisolone (1 g/daily) therapy was administered for 5 days, followed by oral prednisone (1 mg/kg), slowly tapered down during the next 3 months. During the immunosuppressive treatment, our patient had a verified septic state but was successfully treated with intravenous antibiotic therapy. The follow-up MR examination one month after treatment initiation showed mild progression of the WMH lesions with the persistence of previously noted microbleeds (Figure 3 and Figure 4). Although her neurological examination was without focal neurological deficit, the patients was still complaining about the presence of headaches and her control neuropsychological assessment was mildly improved from the initial mini-mental state examination (MMSE) 22/30 to MMSE 23/30. Thus, treatment with oral prednisone (10 mg/daily) was reintroduced. The third follow-up MR examination after six months showed resolutions of the WMH lesions (not shown), while her neuropsychological assessment was further improving.

### 2.3. Third Case Description

**Figure 5:**
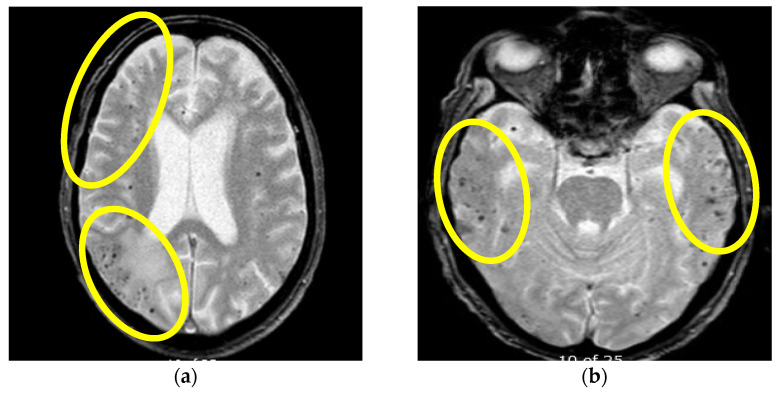
MR axial T2*W sequence (**a**,**b**) showing multilocal punctiform signal hypointensities located diffusely in the subcortex and periventricular posterior region (lobar distribution). Yellow circles in (**a**) are showing multiple multifocal punctiform hypointensities dominantly right frontotemporally and occipitally, and in (**b**) bitemporal multifocal hypointensities (Patient No. 3).

A 65-year-old male patient, with a history of acute myocardial infarction 2 years prior to this hospitalization and essential arterial hypertension, was admitted due to the sudden onset of severe headache and difficulty in speech. Simultaneously, high blood pressure levels were also registered (up to 200/120 mmHg). Upon admission, urgent brain CT examination showed the presence of cortical ischemic leukoencephalopathy together with hypodense vasogenic edema of the right hemisphere, compressing the left lateral brain chamber, as well as a calcified midsagittal meningioma in the frontal lobe. His physical examination did not reveal any unusual findings, whilst the neurologic examination showed a central lesion in the left seventh cranial nerve, dysarthric speech, and mild left-sided spastic hemiparesis with gait ataxia. During the next 24 intrahospital hours, the patient became agitated and spatiotemporally disoriented, with no resolution of the focal neurological signs after the patient’s blood pressure stabilization (initial blood pressure of 200/120 mmHg was lowered to 160/90 mmHg with oral and IV antihypertensive therapy and with diuretics). Standard urine and serum (complete blood count, biochemical, hormonal, vitamin, and tumor status) laboratory results were normal. The results of complete microbiologic and systemic connective tissue disorder testing were normal, while immuno-serologic testing showed the presence of isolated positive nucleoplasmic homogenic antinuclear antibodies (ANA) with a score of 1:320. Chest X-ray and abdominal ultrasound examination did not show any impairment. The results of the lumber puncture showed low glycose levels and elevated protein levels in the CSF (glucose level was 2.2 and proteins 0.49 g/L) together with a normal CSF cell count. All microbiologic, immunological, and sediment CSF investigations were normal. Brain MR revealed parieto-occipito-temporally right, cortically, subcortically, and periventricular confluent zones of T2W/FLAIR hyperintensities of signal with a regional compressive effect on the ventricles. A number of zones of the same MR characteristics are located temporally left in the subcortex in the region of the middle temporal gyrus. Thus, multiple supratentorial and infratentorial punctiform lesions with signal hyperintensity in T2* are located diffusely in the cortex and subcortex including the basal ganglia and brain stem (Figure 5). The prior description revealed the presence of multiple areas of hemoglobin debris with a distribution that corresponds to CAA and merged asymmetric hemispheric WMH lesions, while CT angiography with CT venography examination findings were unremarkable.

Because of high clinical suspicion of probable CAA-rI, the treatment with pulse methylprednisolone therapy (1 g/daily) for 5 days was initiated, followed by oral prednisone (1 mg/kg) and azathioprine introduction (50 mg/day, as a corticosteroid sparing agent). The patient’s condition significantly improved, and his control brain MR evaluation showed partial regression in the initial findings. However, the patient developed an acquired intrahospital gastrointestinal infection with *Clostridium difficile* five days after starting azathioprine therapy and his somatic state, further complicated with respiratory insufficiency, and had to be intubated and connected to mechanical ventilation for a period of 10 days in the intensive care unit. Meanwhile, the doses of both immunosuppressive treatments were significantly reduced. Thus, the patient developed an epileptic seizure manifesting as myoclonus in the region of the left face and left arm, which rapidly progressed into a series of focal epileptic seizures despite antiepileptic treatment introduction (levetiracetam 3 × 1000 mg, midazolam 30 mg IV). A standard electroencephalogram evaluation showed periodic lateralized epileptiform discharges over the right central parietal region (one per second) and were consistent with epilepsia partialis continua. Control MR imaging showed stationary findings where a partial regression of initial WMH lesions was described. Unfortunately, the patient’s somatic state declined further due to systemic infection leading to respiratory insufficiency followed by cardiac arrest in the next 24 h, which resulted in a lethal outcome.

The main clinical, neuroradiological, pathological and treatment outcome data about CAA-rI in to-date literature compared with findings in our patients are presented in Table 1.

## 3. Discussion

Our three reported cases represent patients who fulfilled the diagnostic criteria for probable CAA-rI (all five points accounted for probable CAA-rI) [6,11], since a brain biopsy could not be established due to technical reasons. However, most of the recent publications frequently presented patients diagnosed with probable or possible CAA-rI [12]. Thereby, the urgent need for noninvasive diagnostic criteria was once again underlined, with the final goal of avoiding the potential risks during brain biopsy in a certain number of patients [5,12]. In addition, Chung et al. suggested that early immunosuppressive treatment should be initiated in patients who did not undergo brain biopsy but have clear clinic-radiological suspicion of CAA-rI [11]. In accordance with these findings, all our patients had a satisfying treatment response within 3 weeks, which further supported the diagnosis of CAA-rI and made the brain biopsy easier to postpone, or even not needed.

The diagnosis of CAA-rI heavily relies on non-specific but typical neuroimaging features [24]. On the other hand, the differential diagnosis of CAA-rI comprises a spectrum of disorders, mostly including other immune-mediated brain diseases, infections, malignancies, and hypertensive encephalopathy [12]. However, in up-to-date literature, only a few case series have so far focused on the radiological and clinical correlative findings of CAA-rI [5,7]. A recently conducted meta-analysis has shown that cognitive decline was the most prevalent clinical feature, while MR findings of hyperintense white matter lesions and lobar cerebral microbleeds were most frequently observed in CAA-rI [4]. On the other hand, a recent paper by Sharma et al. provided in detail information on other causes and mimics of neuroradiological findings in CAA-rI [13]. For instance, it was observed that diffuse lobar vasogenic edema, as seen in our or first and third patient, may mimic low-grade infiltrative gliomas [6]. However, brain MR findings in our patients showed distinct characteristics for CAA-rI, where predominantly asymmetric and confluent T2 WM hyperintensities were found in all three of our patients, with two patients having imaging descriptions of cerebral vasogenic edema. Furthermore, all three of our patients had cerebral microbleeds on T2* or susceptibility-weighted imaging (SWI), which are seen in more than 70% of CAA-rI even during the initial disease course [6]. Findings in all our patients have further underlined the asymmetric presentation of several radiographic findings of CAA-rI and its focal impact on the brain, as reported in the up-to-date literature [14]. Nevertheless, previous experience showed that although CAA-rI has varying imaging appearances, the identification of microbleeds in elderly patients presenting with infiltrative white matter process or prominent leptomeningeal enhancement is highly suggestive of this inflammatory type of CAA [6]. Another significant feature of CAA-rI is the frequent reversibility of MR changes, which was more frequently observed in patients who underwent immunosuppressive treatment. For instance, Regenhardt and colleagues have reported that up to 70% of CAA-rI patients had radiographic improvement, 31% had radiographic worsening, and 2% of patients had a static radiographic course [14]. Thus, it seems that both initial and follow-up MR findings of our patients could fit in this heterogenous spectrum of radiological changes in CAA-rI.

The main clinical, diagnostic, and neuroradiographic differences between CAA-rI and ABRA should be especially addressed. For instance, severe headaches and seizures were more frequently observed patients with CAA-rI, while patients diagnosed with ABRA typically presented with focal neurological deficits and cognitive decline [3]. On the other hand, no significant CSF and imaging findings were observed between these two ICAA disorders [3]. Thus, it might be concluded that all these differences seem not to be so dramatic and that further follow-up studies are needed. When compared to patients with primary angiitis of the central nervous system (PACNS) (another primary inflammatory vascular condition), who were significantly younger at disease onset, both CAA-rI and ABRA are most frequently observed in patients who were older than 60 years [3].

The clinical heterogeneity of CAA-rI is clearly presented in all our patients. In the first patient, an impaired state of consciousness with focal neurological signs was predominantly observed, while the second patient mostly complained about a severe intensity headache and had significant behavioral changes. Left-sided spastic hemiparesis and focal seizures, later complicated with focal status epilepticus, were prominent clinical CAA-rI features of the third patient. To our knowledge, till date, there are only two papers, apart from ours, which described the co-occurrence of status epilepticus and CAA-rI presentation [15,16], but none of them presented as epilepsia partialis continua. However, a recent review underlined that cognitive decline, which was present at an early disease stage in all our patients, was the most common clinical manifestation of this disease [4]. Unfrequently, patients with CAA-rI are presented with rapid cognitive decline or even dementia, both of which are symptoms that arise over weeks to months [17,18]. It seems that the presence and etiology of a preexisting cognitive impairment influences the type of the presented cognitive disfunction in patients with CAA-rI. Indeed, patients with CAA-rI have a more severe amyloid load on PET, when compared to CAA [19]. However, memory impairment and confusion/attention deficits are the most frequent de novo types of cognitive decline in CAA-rI patients [17], which was in accordance with findings in our patients where memory dysfunction, attention and executive deficits were observed. Surprisingly, Plotzker et al. showed that treatment outcome in CAA-rI patients was not influenced by their baseline cognitive status [17], which might further motivate and early immunosuppressive therapeutic intervention.

Although Salvarani and colleagues have observed that CAA-rI was more closely related to primary CNS vasculitis than to CAA without inflammation [6], in most of the cases reported until date, angiography examination findings were normal [6,9]. This is in accordance with the investigation results in our patients where both angiography and immuno-serologic examination findings were unremarkable. On the other hand, a recent case report presented a patient with CAA who developed probable CAA-rI/ABRA several months after an acute ischemic stroke, suggesting ischemia with blood–brain barrier disruption as a potential risk factor [20]. Most of the reported patients in previous literature had elevated CSF protein levels [21,25], which is in accordance with findings in all our patients. However, elevated CSF protein levels are not specific nor sensitive for the diagnosis [26]. Although oligoclonal bands are typically not present in this disease, few individual case reports show positive OB findings in patients with CAA-rI [15]. Interestingly, one paper suggested that OB can also occasionally be found in patients with Hepatitis B infection [27], which was noted in our first patient. However, our first patient had initially positive oligoclonal bands in serum and CSF, but his control CSF evaluation was completely normal, which was also occasionally seen in the up-to-date literature and could at least partially be explained by the effect of immunosuppressive therapy or the natural course of this disease [28,29]. At the moment of diagnostic evaluation and to date follow-up assessment, our patients did not fulfill the diagnostic criteria for any other differential diagnosis. However, misleading findings, such as a verified hepatitis B viral infection in the first case and mostly isolated (exclusive headache) rapid cognitive decline of the second patient, were an initial burden in establishing the reliable diagnosis.

Considering CAA-rI as an inflammatory vasculopathy, different immunosuppressive agents are often introduced (most commonly corticosteroids, cyclophosphamide, mycophenolate mofetil and azathioprine), but with various clinical and radiologic outcome reported [7,9,12,16,26]. On the other hand, having in mind that several CAA-ri cases with spontaneous recovery we reported in previous literature, it seems that a clear association between disease outcome and immunosuppressive treatment is still poorly understood. For instance, amyloid-related inflammation may positively respond to immunosuppressive treatment, but the basic vascular pathology of CAA will remain unchanged and represents a significant continuous risk for further vascular events [22]. However, recent data suggested that early immunosuppressive treatment introduction in CAA-rI patients could have long-term benefits and modulate the disease course and outcome [9,14].

Previous data have shown that a favorable outcome was seen in between 42% and 75% of CAA-rI patients [10,11]. In addition, a more recent systematic review of both pathologic variants of CAA-rI showed that about half of all patients were asymptomatic or with mild neurological sequelae after a two-year follow-up period [4]. In contrast with these findings, several reports of death or significant disability in up to 60% of treated CAA-rI patients further emphasize the persistent heterogeneity of the disease course and outcome in these patients [4,9,23]. It seems that one of the main predictors of poor outcome in CAA-rI are infectious complications of immunosuppressive therapy [21]. In addition to these findings, one of two of our patients who had a verified septic state during the mid-hospital course and while on immunosuppressive therapy, had an unfavorable outcome. However, the overall up-to-date data about prognostic factors and outcome predictors of CAA-rI are scarce, but Coluette et al. have reported that the initial patient’s clinical status, occurrence of intracerebral hemorrhage, and a high relapse rate of CAA-rI were predictive of a poorer outcome [21]. Different recurrence rates of CAA-rI are present in up-to-date literature, and relapse during immunosuppressive drug dose decrease or after drug withdrawal can be seen in these patients [30], as it was noted in one our patient after dose reduction. With ongoing immunosuppressive therapy, the first patient was completely recovered at discharge, while the second patent started to have a mild cognitive improvement but was without any previously noted clinical impairment, and the third patient eventually died despite aggressive antiepileptic treatment.

### Possible Future Perspectives

However, despite the up-to-date established diagnostic criteria for CAA-rI, this disorder is still insufficiently recognized and treated [1,4], and the definitive diagnosis can be established only using brain biopsy. Thus, other genetic, immunologic, and CSF biomarkers are urgently needed. For instance, the significance of amyloid-β biomarkers in serum/CSF is rapidly expanding in other amyloidopathies (such as Alzheimer’s disease and even CAA) [31], but their role in CAA-rI is still insufficiently investigated. Moreover, other brain imaging biomarkers, such as amyloid PET, might also provide further diagnostic evidence of CAA-rI and serve as tools for evaluating treatment efficacy. However, these diagnostic tools are still unavailable in most of the countries, and further investigation is clearly needed. Certainly, new therapeutic strategies for this immune-mediated disease need to be developed, in parallel.

One of the main limitations of our study is the fact that the diagnosis of CAA-rI was not confirmed via brain biopsy, which is still the “golden standard”. However, typical initial clinical and neuroimaging features of CAA-rI, combined with clear clinical and neuroradiographic response to immunosuppressive therapy, was supportive of probable CAA-rI. In addition, patient’s long-term follow-up data would also be of further significance. At least, our review was mainly focused on up-to-date literature data, which are clearly relevant to our case reports.

## 4. Conclusions

Herein, we describe three patients with probable CAA-rI who presented with a significant heterogeneity of both clinical and neuroradiological features. Clinicians who are considering the diagnosis of CAA-rI should attribute special attention to patients with acute/subacute emergence of cognitive/behavioral changes or decreased consciousness, followed by the appearance of different focal neurologic deficits, which could not be explained by other possible causes. When this “clinical mosaic of CAA-rI” is further accompanied with typical neuroradiographic findings such as cerebral microbleeds and asymmetric WMH lesions, a possible/probable diagnosis of CAA-rI must be considered. In addition, it is also paramount to consider other less frequent clinical presentations of CAA-rI such as epilepsia partialis continua. Finally, prompt introduction to immunosuppressive therapy must be considered since early treated patients might achieve a significant reversion of the clinico-radiological dissemination of CAA-rI, which is the main prerequisite of better disease outcome and quality of life.

## Figures and Tables

**Table 1:** Summarized main clinical, neuroradiological, pathological and treatment outcome data about CAA-rI compared with findings in our patients.

	Literature Data [1,3,4,5,6,7,8,9,10,11,12,13,14,15,16,17,18,19,20,21,22,23]	Our Case Reports
Main clinical features (Including atypical features)	Cognitive decline Behavioral decline Encephalopathy Focal neurological deficit Headache Seizures Stroke-like episodes	Acute/subacute cognitive and behavioral decline Decreased consciousness (without proven encephalopathy) Focal neurological deficit Headache Seizures (including focal epileptic status)
Neuroradiographic features	Intracerebral hemorrhage T2/fluid-attenuated inversion recovery-hyperintense white matter lesions Meningeal gadolinium enhancement Lobar cerebral microbleeds Cortical superficial siderosis	Asymmetric WMH lesions, Cerebral microbleeds
Core pathological findings	Amyloid beta deposition and non-destructive perivascular inflammation	Not applicable
Main immunosuppressive treatment steps	First-line treatment: corticosteroid therapy * Second-line treatment: cyclophosphamide, mycophenolate mofetil, azathioprine, methotrexate * Third-line treatment: intravenous immunoglobulins *	Not applicable
Treatment response/prognosis	Mostly positive (clinical improvement with at least partial regression of MR changes)	Positive in two cases and negative (death) in one case

* In parallel with appropriate symptomatic therapy.

## Data Availability

The data that support the findings of this study are available on request from the corresponding author. The data are not publicly available due to privacy and ethical restrictions.

## Abbreviations

Activated partial thromboplastin time (aPTT), Activated Protein C Sensitivity Ratio (APCR.Ratio), Angiotensin converting enzyme (ACE), Antineutrophil Cytoplasmic Antibodies (ANCA), Antinuclear antibody (ANA), Antinuclear antibody-HEp-2 (ANA-HEp2), Anti-mitochondrial M2 antibody (AMA M2), Amyloid β-related angiitis (ABRA), *Apolipoprotein E* (*ApoE*), Carcinoembryonic antigen (CEA), Central nervous system (CNS), Cerebral amyloid angiopathy-related inflammation (CAA-rI), Cancer antigen (CA), Cerebral amyloid angiopathy (CAA), Cerebrospinal fluid (CSF), Cytokeratin 19 Fragment (CYFRA 21-1), Deoxyribonucleic acid (DNA), Epstein-Barr virus (EBV), Extractable Nuclear Antigen screen (ENA Screen), Fluid-attenuated inversion recovery (FLAIR), Hepatitis B virus (HBV), Hepatitis C virus (HCV), Human immunodeficiency virus (HIV), Herpes simplex virus (HSV), Human polyomavirus (JCV), Inflammatory cerebral amyloid angiopathy (ICAA), Intravenous (IV), Lupus Anticoagulant 1 (LA1), Magnetic Resonance (MR), Mini–mental state examination (MMSE), MR angiography (MRA), *Neurogenic locus notch homolog protein 3* (*NOTCH3*), Oligoclonal bands (OCB), Primary angiitis of the central nervous system (PACNS), Positron emission tomography (PET), Real time-polymerase chain reaction (PCR), Rontgen radiation (X-ray), Signal intensity (SI), The Glasgow Coma Scale (GCS), Thyrotropin hormone (TSH), Free thyroxine (FT4), Free tri-iodothyronine (FT3) White matter hyperintensities (WMH), Venereal Disease Research Laboratory (VDRL), Von Willebrand factor (VWF).

## References

[1] Chwalisz B.K. (2021). Cerebral amyloid angiopathy and related inflammatory disorders. J. Neurol. Sci..

[2] Makarewicz K.A., Zaryczańska K., Machowska-Sempruch K., Bajer-Czajkowska A., Gołofit P., Gabrysz-Trybek E., Nowacki P. (2019). Cerebral amyloid angiopathy-related inflammation (CAARI): Case report. Folia Neuropathol..

[3] Ekkert A., Šaulytė M., Jatužis D. (2022). Inflammatory Disorders of the Central Nervous System Vessels: Narrative Review. Medicina.

[4] Theodorou A., Palaiodimou L., Malhotra K., Zompola C., Katsanos A.H., Shoamanesh A., Boviatsis E., Dardiotis E., Spilioti M., Sacco S. (2023). Clinical, Neuroimaging, and Genetic Markers in Cerebral Amyloid Angiopathy-Related Inflammation: A Systematic Review and Meta-Analysis. Stroke.

[5] Wu J.J., Yao M., Ni J. (2021). Cerebral Amyloid Angiopathy-Related Inflammation: Current Status and Future Implications. Chin. Med. J..

[6] Salvarani C., Morris J.M., Giannini C., Brown R.D., Christianson T., Hunder G.G. (2016). Imaging Findings of Cerebral Amyloid Angiopathy, Aβ-Related Angiitis (ABRA), and Cerebral Amyloid Angiopathy-Related Inflammation: A Single-Institution 25-Year Experience. Medicine.

[7] Auriel E., Charidimou A., Gurol M.E., Ni J., Van Etten E.S., Martinez-Ramirez S., Boulouis G., Piazza F., DiFrancesco J.C., Frosch M.P. (2016). Validation of Clinicoradiological Criteria for the Diagnosis of Cerebral Amyloid Angiopathy–Related Inflammation. JAMA Neurol..

[8] Theodorou A., Palaiodimou L., Safouris A., Kargiotis O., Psychogios K., Kotsali-Peteinelli V., Foska A., Zouvelou V., Tzavellas E., Tzanetakos D. (2022). Cerebral Amyloid Angiopathy-Related Inflammation: A Single-Center Experience and a Literature Review. J. Clin. Med..

[9] Mendonça M.D., Caetano A., Pinto M., Cruz e Silva V., Viana-Baptista M. (2015). Stroke-Like Episodes Heralding a Reversible Encephalopathy: Microbleeds as the Key to the Diagnosis of Cerebral Amyloid Angiopathy-Related Inflammation-A Case Report and Literature Review. J. Stroke Cerebrovasc. Dis..

[10] Sowanou A.V., Ungureanu A., Paulin M. (2022). Cerebral Amyloid Angiopathy Related Inflammation with Leptomeningeal Involvement: A Case Report and Review of the Literature. Acta Neurol. Belg..

[11] Chung K.K., Anderson N.E., Hutchinson D., Synek B., Barber P.A. (2011). Cerebral Amyloid Angiopathy Related Inflammation: Three Case Reports and a Review. J. Neurol. Neurosurg. Psychiatry.

[12] Kirshner H.S., Bradshaw M. (2015). The Inflammatory Form of Cerebral Amyloid Angiopathy or “Cerebral Amyloid Angiopathy-Related Inflammation” (CAARI). Curr. Neurol. Neurosci. Rep..

[13] Sharma R., Dearaugo S., Infeld B., O’Sullivan R., Gerraty R.P. (2018). Cerebral amyloid angiopathy: Review of clinico-radiological features and mimics. J. Med. Imaging Radiat. Oncol..

[14] Regenhardt R.W., Thon J.M., Das A.S., Thon O.R., Charidimou A., Viswanathan A., Gurol M.E., Chwalisz B.K., Frosch M.P., Cho T.A. (2020). Association between immunosuppressive treatment and outcomes of cerebral amyloid angiopathy-related inflammation. JAMA Neurol..

[15] Tolchin B., Fantaneanu T., Miller M., Helgager J., Lee J.W. (2016). Status epilepticus caused by cerebral amyloid angiopathy-related inflammation. Epilepsy Behav. Case Rep..

[16] Watanabe Y., Kuroda H., Nishiyama S., Kobayashi J., Jin K., Aoki M. (2017). Disseminated cerebral amyloid angiopathy-related inflammation manifesting as non-convulsive status epilepticus. Neurol. Clin. Neurosci..

[17] Plotzker A.S., Henson R.L., Fagan A.M., Morris J.C., Day G.S. (2021). Clinical and paraclinical measures associated with outcome in cerebral amyloid angiopathy with related inflammation. J. Alzheimers Dis..

[18] Day G.S. (2022). Rapidly progressive dementia. Continuum.

[19] Renard D., Tatu L., Collombier L., Wacongne A., Ayrignac X., Charif M., Boukriche Y., Chiper L., Fourcade G., Azakri S. (2018). Cerebral amyloid angiopathy and cerebral amyloid angiopathy-related inflammation: Comparison of hemorrhagic and DWI MRI features. J. Alzheimers Dis..

[20] Singh B., Lavezo J., Gavito-Higueroa J., Ahmed F., Narasimhan S., Brar S., Cruz-Flores S., Kraus J. (2022). Updated Outlook of Cerebral Amyloid Angiopathy and Inflammatory Subtypes: Pathophysiology, Clinical Manifestations, Diagnosis and Management. J. Alzheimers Dis. Rep..

[21] Coulette S., Renard D., Lehmann S., Raposo N., Arquizan C., Charif M., Boukriche Y., Gaillard N., Thouvenot E. (2019). A Clinico-Radiological Study of Cerebral Amyloid Angiopathy-Related Inflammation. Cerebrovasc. Dis..

[22] Reisz Z., Troakes C., Sztriha L.K., Bodi I. (2022). Fatal Thrombolysis-Related Intracerebral Haemorrhage Associated with Amyloid-β-Related Angiitis in a Middle-Aged Patient—Case Report and Literature Review. BMC Neurol..

[23] Castro Caldas A., Silva C., Albuquerque L., Pimentel J., Silva V., Ferro J.M. (2015). Cerebral amyloid angiopathy associated with inflammation: Report of 3 cases and systematic review. J. Stroke Cerebrovasc. Dis..

[24] Cancelloni V., Rufa A., Battisti C., De Stefano N., Mastrocinque E., Garosi G., Venezia D., Chiarotti I., Cerase A. (2022). Diagnosis, Treatment, and Follow-Up of Patients with Cerebral Amyloid Angiopathy-Related Inflammation. Neurol. Sci..

[25] Corovic A., Kelly S., Markus H.S. (2018). Cerebral Amyloid Angiopathy Associated with Inflammation: A Systematic Review of Clinical and Imaging Features and Outcome. Int. J. Stroke.

[26] Chen D., Roytman M., Kirou K.A., Navi B.B., Schweitzer A.D. (2022). A Case of Inflammatory Cerebral Amyloid Angiopathy after Ischemic Stroke—A Potential Risk Factor Related to Blood-Brain Barrier Disruption. Clin. Imaging.

[27] Papadopoulos N.M., Tsianos E.V., Costello R. (1990). Oligoclonal immunoglobulins in serum of patients with chronic viral hepatitis. J. Clin. Lab. Anal..

[28] Traschütz A., Tzaridis T., Penner A.H., Kuchelmeister K., Urbach H., Hattingen E., Heneka M.T. (2015). Reduction of Microbleeds by Immunosuppression in a Patient with Aβ-Related Vascular Inflammation. Neurol. Neuroimmunol. Neuroinflamm..

[29] Trbojevic-Cepe M. (2004). Detection of Oligoclonal Ig Bands: Clinical Significance and Trends in Methodological Improvement. EJIFCC.

[30] Danve A., Grafe M., Deodhar A. (2014). Amyloid beta-related angiitis—A case report and comprehensive review of literature of 94 cases. Semin. Arthritis Rheum..

[31] Charidimou A. (2022). Cerebrospinal Fluid Biomarkers for Cerebral Amyloid Angiopathy Diagnosis. J. Alzheimers Dis..

